# Mesenchymal Stem Cell-Derived Exosomes Promote Recovery of The Facial Nerve Injury through Regulating Macrophage M1 and M2 Polarization by Targeting the P38 MAPK/NF-Κb Pathway

**DOI:** 10.14336/AD.2023.0719-1

**Published:** 2024-04-01

**Authors:** Ruoyan Xue, Mengyao Xie, Zhiyuan Wu, Shu Wang, Yongli Zhang, Zhijin Han, Chen Li, Qi Tang, Liping Wang, Di Li, Shihua Wang, Hua Yang, Robert Chunhua Zhao

**Affiliations:** ^1^Department of Otolaryngology, Peking Union Medical College Hospital, Chinese Academy of Medical Sciences and Peking Union Medical College, Beijing, China.; ^2^Chinese Academy of Medical Sciences and Peking Union Medical College, Beijing, China.; ^3^Institute of Basic Medical Sciences Chinese Academy of Medical Sciences, School of Basic Medicine Peking Union Medical College, Peking Union Medical College Hospital, Center of Excellence in Tissue Engineering Chinese Academy of Medical Sciences, Beijing Key Laboratory, Beijing, China.; ^4^School of Life Sciences, Shanghai University, Shanghai, China.

**Keywords:** facial nerve injury, mesenchymal stem cell, exosome, macrophage polarization

## Abstract

Facial nerve (FN) injury seriously affects human social viability and causes a heavy economic and social burden. Although mesenchymal stem cell-derived exosomes (MSC-Exos) promise therapeutic benefits for injury repair, there has been no evaluation of the impact of MSC-Exos administration on FN repair. Herein, we explore the function of MSC-Exos in the immunomodulation of macrophages and their effects in repairing FN injury. An ultracentrifugation technique was used to separate exosomes from the MSC supernatant. Administrating MSC-Exos to SD rats via local injection after FN injury promoted axon regeneration and myelination and alleviated local and systemic inflammation. MSC-Exos facilitated M2 polarization and reduced the M1-M2 polarization ratio. miRNA sequencing of MSC-Exos and previous literature showed that the MAPK/NF-κb pathway was a downstream target of macrophage polarization. We confirmed this hypothesis both *in vivo* and *in vitro*. Our findings show that MSC-Exos are a potential candidate for treating FN injury because they may have superior benefits for FN injury recovery and can decrease inflammation by controlling the heterogeneity of macrophages, which is regulated by the p38 MAPK/NF-κb pathway.

## INTRODUCTION

The facial nerve (FN) is crucial for both social and physiological processes. FN injury may result in both physical and mental incapacitation [1, 2]. Even with the finest surgical restoration, functional outcomes are still suboptimal in cases of severe FN injuries that do not heal on their own [3]. The long-term persistence of neuroinflammation is thought to prevent repair and regeneration. Recent studies have revealed that macrophage polarization is crucial to the activation and control of neuroinflammation during peripheral nerve damage and repair [4, 5]. Classically activated macrophages (M1) and alternatively activated macrophages (M2) are two macrophage types [4, 6]. The former provides an environment that is proinflammatory and removes myelin debris after damage [7]. The latter becomes more common with time. M2 macrophages are essential for the regeneration process because they secrete a variety of growth factors and anti-inflammatory cytokines [8]. Therefore, modulating the equilibrium between M1 and M2 macrophages represents a promising therapeutic strategy for treating FN injury; this approach couple promote earlier and greater infiltration of M2 macrophages as well as reducing harmful excessive neuroinflammation.

The multilineage differentiation capacity and immunosuppressive qualities of mesenchymal stem cells (MSCs) make them potentially useful as cell therapies in regenerative medicine, including for the treatment of FN damage [9, 10]. There is increasing evidence that paracrine secretion contributes to the effectiveness of MSC-based therapeutic approaches, particularly in terms of their ability to control the immunological microenvironment [11-13]. The role of exosomes as key regulatory mediators is increasingly being recognized since they are a product of paracrine secretion. Exosomes are nanoscale extracellular vesicles that form when multivesicular bodies (MVBs) fuse with the cellular membrane. These vesicles have a particular cargo composition that includes proteins and microRNAs (miRNAs), which facilitate intercellular communication between cells [14]. Recent research has shown that MSC-derived exosomes (MSC-Exos) have therapeutic capabilities that are comparable to those of their parent MSCs. Moreover, MSC-Exos provide numerous noteworthy advantages over cell treatment, including low immunogenicity, simple storage, and good biosafety [15]. MSC-Exos are frequently researched as cell-free therapeutic agents for the treatment of spinal cord and brain injuries since they have been demonstrated to have significant promise in anti-inflammation and injury repair [11, 16]. It has also been discovered that their therapeutic effect is related to the encouragement of microglial /macrophage polarization to reduce inflammation [17, 18]. It is unclear, however, whether MSC-Exos could significantly contribute to axonal outgrowth by suppressing M1 polarization and encouraging M2 polarization to foster an anti-inflammatory environment in FN injury.

Consequently, the purpose of this work was to determine whether MSC-Exos might block M1 polarization, promote M2 polarization, and accelerate FN injury recovery. Moreover, we used miRNA sequencing and bioinformatic analysis to study the underlying molecular processes. Our results suggest that the influence of MSC-Exos on macrophages was controlled by mediating the p38MAPK/NF-κb signaling pathway. Herein, we present the first data demonstrating the use of MSC-Exos in treating FN injury; treatment reduced inflammation and stimulated regeneration by controlling macrophage heterogeneity, indicating that MSC-Exos are a potential candidate for treating FN damage.

## MATERIALS AND METHODS

### Cell culture

The Peking Union Medical College Ethics Committee and the Chinese Academy of Medical Sciences authorized the methods used to harvest human adipose tissues. Human adipose-derived mesenchymal stem cells (hAdMSCs) were collected from healthy adult donors’ lipoaspirate human adipose tissue and culture-expanded as previously described.

Human monocytic THP-1 cells (BeNa Culture Collection, BNCC, Beijing, China) were maintained in Roswell Park Memorial Institute (RPMI 1640, HyClone) culture medium containing 10% fetal bovine serum (FBS, Gibco). After treatment with 100 ng of phorbol 12-myristate 13-acetate (PMA; Sigma–Aldrich, P8139) for 48 h, macrophages were obtained through the transformation of THP-1 monocytes.

A total of 100 ng/mL lipopolysaccharide (LPS, Sigma–Aldrich, L6529) was used to treat macrophages to induce an inflammatory environment; the cells were further incubated with MSC-Exos, PBS, MSC-Exos+ciglitazone (HY-W011220, MCE), or MSC-Exos+NF-κΒ activator 1 (HY-134476, MCE) for 48 h. Cells not treated with LPS or MSC-Exos were considered the M0 phenotype negative control. After a 48-h incubation, the supernatants were collected to measure the levels of specific proteins using an enzyme-linked immunosorbent assay, and we collected cells for further RNA isolation and flow cytometry analysis.

The cells were kept at 37 °C in a humidified incubator (5% CO_2_) and were routinely passaged by trypsinization when they reached 90% confluency.

### Characterization of hAdMSCs

Analyses of the immunophenotype of hAdMSCs were conducted as previously described [19, 20]. After being washed, hAdMSCs were subjected to incubation for half an hour at 4 °C with primary antibodies against human, including CD34-PE (cat#550761, BD Biosciences), CD44-PE (cat# 559942, BD Biosciences), CD29-PE (cat#556049, BD Biosciences), CD73-PE (cat# 550257, BD Biosciences), CD90-PE (cat# 555596, BD Biosciences), CD45-PE (cat# 560975, BD Biosciences), CD106-PE (cat# 561679, BD Biosciences), CD105-PE (cat# 560839, BD Biosciences), CD206-FITC (cat#5 51135, BD Biosciences) and HLA-DR-PE(cat# 555561, BD Biosciences). Following washing, hAdMSCs were subjected to incubation with secondary antibodies for half an hour at 4 °C. MSCs were then examined using FACSCalibur (BD Biosciences) and the FlowJo program (FlowJo, LCC). Moreover, adipogenic and osteogenic differentiation media were used to cultivate the cells. We performed oil red O staining to assess the adipogenic differentiation of cells (Solarbio, China). Alkaline phosphatase (ALP), an early osteogenic marker, and Alizarin Red (ARS), a late osteogenic marker, were used to quantify the osteogenic differentiation of the cells.

### Exosome extraction and characterization

Exosomes were isolated and characterized according to MISEV 2018 guidelines [21]. When the confluency of hAdMSCs was 80-90%, we replaced the culture medium with serum-free medium for another 48 h to prevent vesicle contamination from serum. To eliminate dead cells, we collected the media and subjected it to centrifugation at 800 × g for 5 min and then 3000 × g for 10 min. Following pore polyethersulfone membrane filtering (0.1 mm; Corning) to remove cell debris and large vesicles, a cutoff membrane (100,000 Mw) was used to concentrate the supernatant (CentriPlus-70, Millipore). The supernatant volume was decreased from approximately 250-500 mL to less than 5 mL. A 70Ti Rotor (Beckman Coulter) was used to ultracentrifuge the supernatant at 110,000 × g for two hours at 4 °C. The produced pellets were then resuspended in PBS and ultracentrifuged using a 100 Ti Rotor for 1 h at 110,000 × g at 4 °C (Beckman Coulter). We either stored exosomes at -80 °C or used them immediately for subsequent experiments.

Transmission electron microscopy was used to study exosome morphology. Ten microliters of exosome solution were spread out over a copper mesh and allowed to sit for one minute at room temperature. The exosomes were treated with uranyl acetate solution for 1 min after being cleaned with sterile deionized water. We then dried the sample with incandescent light for two minutes. Using TEM, the copper mesh was examined and photographed by a camera (H-7650, Hitachi Ltd., Tokyo, Japan).

The exosome size distribution and concentration (NTA) were measured using nanoparticle tracking analysis. To assess the size and number of particles separated, exosomes resuspended in PBS were introduced into the ZetaView PMX 110 (Particle Metrix, Meerbusch, Germany) fitted with a 405 nm laser. The frame rate of a 60-second movie was set to 30 frames each second, and NTA software was used to examine the movement of particles in the film (ZetaView 8.02.28).

We utilized Western blotting to measure some exosome surface markers, including Tsg101 (cat#sc-13611, Santa Cruz, CA, USA), Alix (cat#sc-53540, Santa Cruz, CA, USA), HSP70(cat# ab181606, Abcam, USA), and CD81(cat# 27855-1, Proteintech, USA). Calnexin (cat# 10427-2-AP, Promega, Madison, WI) served as a negative marker for exosomes.

### Exosome uptake

MSC-Exos were labeled with CM-DIL red dye (Thermo Fisher) following the manufacturer’s protocol. The labeled exosomes were then supplemented with or without LPS stimulation of M0 macrophages and cocultured for 0 min, 6, 12 or 24 h. Then, 4% formaldehyde was applied to fix the cells, and 0.1% Triton was employed for permeation. We added DAPI dropwise and incubated the cells in the dark for 5 min to stain the nuclei. Fluorescence images were obtained using Zeiss LSM 880 confocal microscopes.

### Flow cytometry analysis

Flow cytometry was performed using standard protocols. An Fc receptor block (BioLegend) was incubated with samples to reduce nonspecific antibody binding for half an hour at 4 °C. Afterward, the cells were incubated for half an hour at 4 °C with fluorochrome-tagged monoclonal antibodies from BioLegend, such as anti-human CD11b APC (cat# 301330), anti-human CD80 PE (cat# 305220), anti-human CD11c APC (cat# 301614), and anti-human CD206 FITC (cat#321110). Cell populations were gated as follows: M1 macrophages (CD11b+CD 80+) and M2 macrophages (CD11b+ CD206+). We performed flow cytometry using a FACSCalibur flow cytometer (BD Bioscience), and FlowJo software (FlowJo, LCC) was used for data analysis.

### Quantitative real-time PCR

Using qRT–PCR, we quantified the relative gene expression of the surface, intracellular, and intranuclear markers defining M1 and M2 macrophages. qRT-PCR was performed according to standard protocols*.* TRIzol reagent (Invitrogen, 10296010) was utilized to extract total RNA from the cells. The PrimeScript RT Reagent Kit (Takara, RR037Q) was adopted for the reverse transcription of the extracted total RNA to obtain complementary DNA (cDNA). cDNA fragments for quantitative PCR were detected using SYBR Green detection reagent (Takara). We assessed relative mRNA expression utilizing the 2-ΔΔCt method and normalized it to the expression of GAPDH. All primers were obtained from Invitrogen Company (Beijing, China).

**Figure 1.**
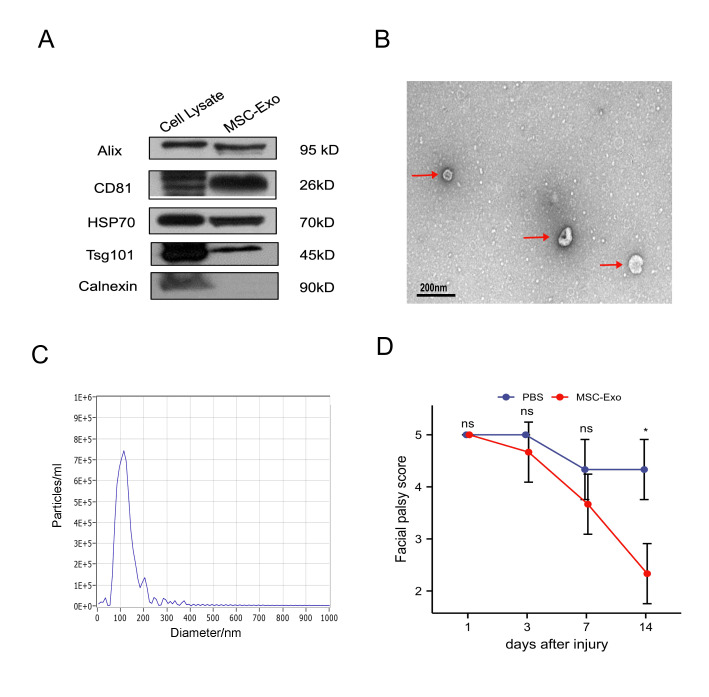
**Characterization of MSC-Exos and behavior assessment after MSC-Exo treatment**. **(A)** Representative images of Western blotting analysis showing the biomarkers of MSC-Exos, including Alix, CD81, HSP70, and Tsg101. Calnexin was used as a negative control. **(B)** Transmission electron microscopy images of MSC-Exos. Red arrows indicate typical cup- or sphere-shaped exosomes (n=3, scale bar=200 nm). **(C)** Nanoparticle tracking analysis reveals the particle distribution of exosomes of various sizes. Within every nanometer diameter set, the value of the ordinate represents the mean particle number. **(D)** Scores in the evaluation of facial muscle function in SD rats during treatment. The total number at each time point is the sum of tip position, vibrissae movement, and blink reflex. The higher the score, the more severe the facial paralysis symptoms are. Facial paralysis is diagnosed when a total score is greater than 3 points. The total scores of the MSC-Exos group were significantly lower than those in the PBS group at day 14 post-surgery. Data are presented as median and IQR (*n* = 3; a non-parametric Mann-Whitney test was performed). *P < 0.05, vs. PBS group; ns, not significant.

**Figure 2.**
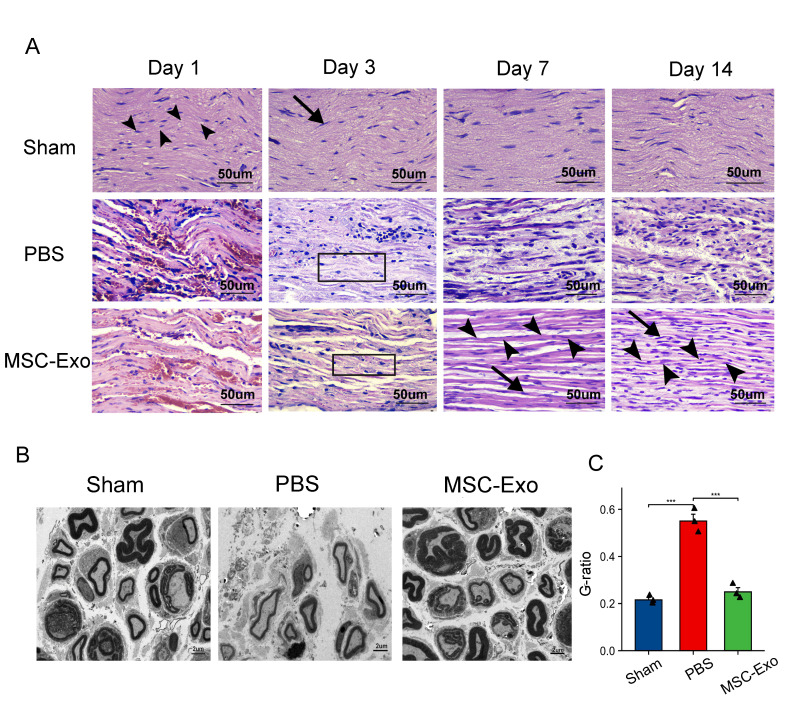
**MSC-Exos promote axonal regeneration and myelination *in vivo***. **(A)** H&E staining images in the sham group, PBS group and MSC-Exo group at days 1, 3, 7 and 14 after injury (n = 3, scale bar=50 µm). Black arrow heads indicate nerve fibers. Black arrows indicate Schwann cells. Black rectangular boxes indicate Wallerian degeneration, considerable myelin sheath disintegration. **(B)** Transmission electron microscopy was performed to observe the regeneration of the myelin sheath at 14 days after injury (n=3, scale bar=2µm). **(C)** Quantification of myelin sheath thickness by G-ratio analysis in B. Data are presented as the mean ± SD (*n* = 3; each black triangle represents one sample). Statistical significance was determined using one-way ANOVA followed by Tukey’s HSD *post hoc* test. ***P < 0.001.

**Figure 3.**
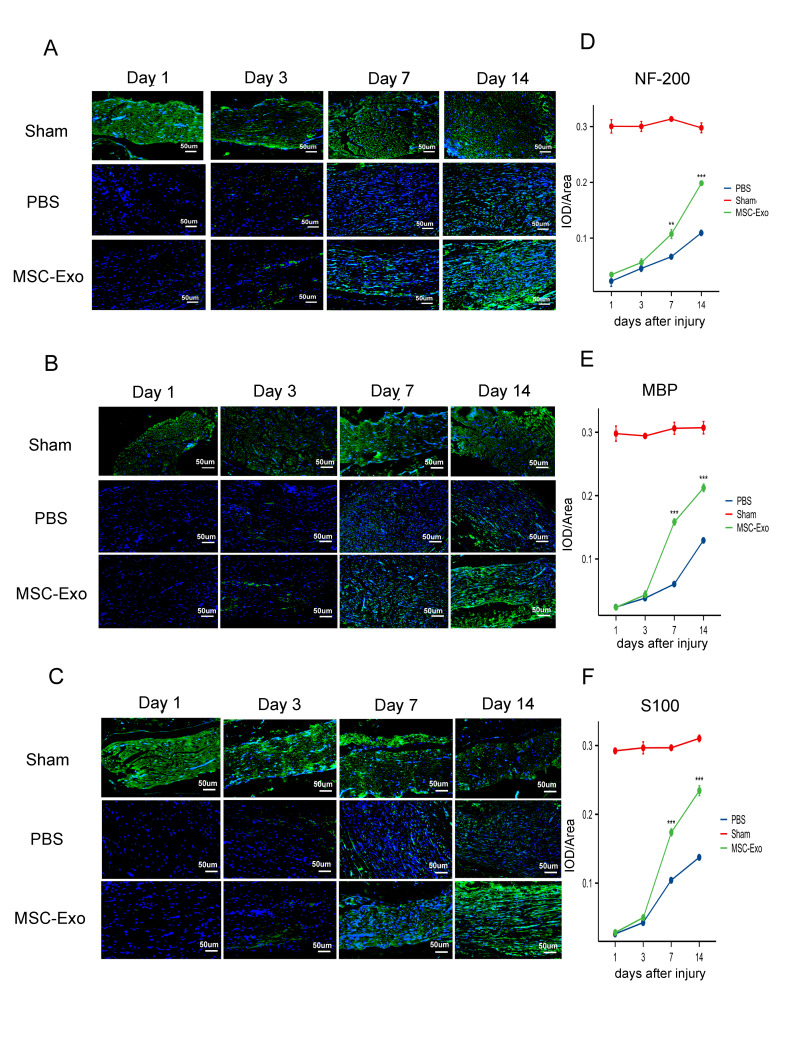
**Immunofluorescence staining of NF200, MBP, and S100**. (A, D) Representative immunofluorescence images and quantification analysis of NF200 expression in facial nerve tissues from the 3 groups at days 1, 3, 7 and 14 after injury (n=3, scale bar=50 µm, green = NF200, blue=DAPI). (B, E) Representative immunofluorescence images and quantification analysis of MBP expression in facial nerve tissues from the 3 groups at days 1, 3, 7 and 14 after injury (n=3, scale bar=50 µm, green =MBP, blue=DAPI). (C, F) Representative immunofluorescence images and quantification analysis of S100 expression in facial nerve tissues from the 3 groups at days 1, 3, 7 and 14 after injury (n=3, scale bar=50 µm, green =S100, blue=DAPI). All data are presented as the mean ± SD. Two-way repeated-measures ANOVA followed by followed by univariate tests of simple main effects with Bonferroni correction for post hoc comparisons. **P < 0.01, ***P < 0.001.

**Figure 4.**
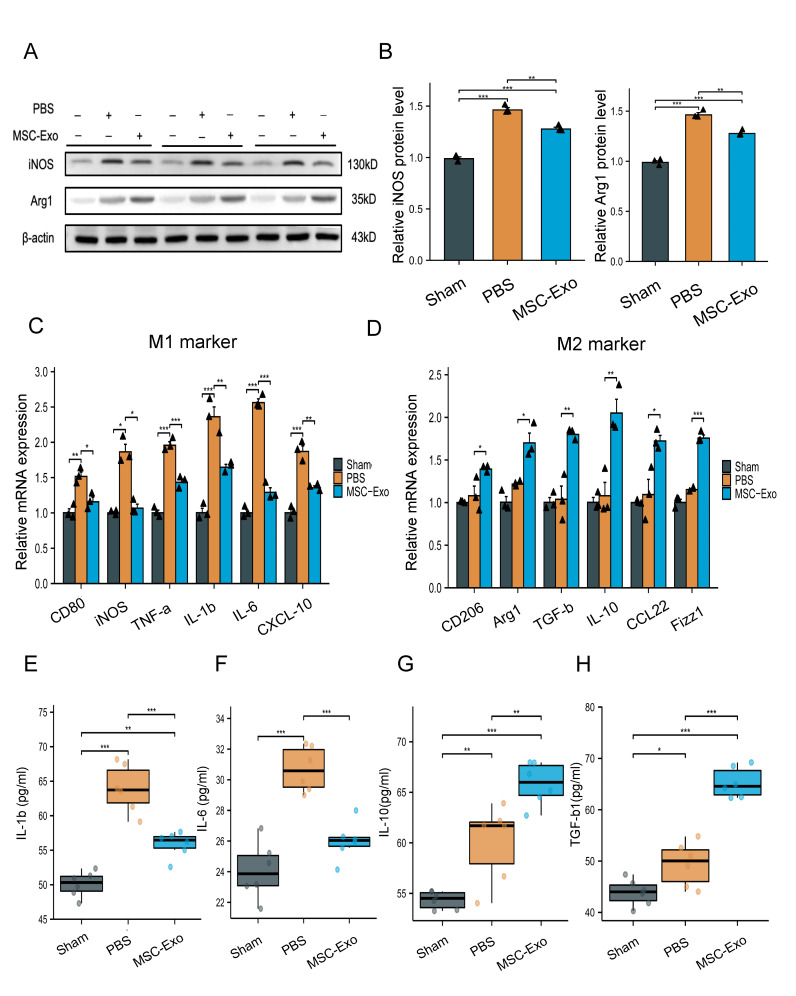
**Effects of MSC-Exos on macrophage polarization following facial nerve injury**. **(A)** Representative Western blotting images to assess the levels of iNOS and Arg1 in the facial nerve of rats treated with PBS or MSC-Exos 7 days after injury (n=3; each black triangle represents one sample). β-Actin was utilized as an internal reference. **(B)** Quantification of band intensities in A (n=3; each black triangle represents one sample). All data are the mean ± SD. Statistical significance was determined using one-way ANOVA followed by Tukey’s HSD *post hoc* test. **P<0.01; ***P<0.001. (C, D) qRT–PCR analysis of M1 marker and M2 marker mRNA levels in the facial nerve of rats treated with PBS or MSC-Exos 7 days after injury (n=3; each black triangle represents one sample). All data are presented as the mean ± SD. Statistical significance was determined using Welch one-way ANOVA with the Games-Howell *post hoc* test for iNOS and Arg1. The other groups were using one-way ANOVA followed by Tukey’s HSD *post hoc* test. *P<0.05; **P<0.01; ***P<0.001. **(E-H)** Cytokine expression of IL-1β (E) and IL-6 (F) in rat serum treated with PBS or MSC-Exos 7 days after facial nerve injury (n= 3; each black triangle represents one sample). Concentration of the c2ytokines IL-10 (G) and TGF-β1 (H) in serum 7 days after injury (n= 3; each black triangle represents one sample). Graphs depict the mean ± SD. Statistical significance was determined using one-way ANOVA followed by Tukey’s multiple comparisons test for multiple groups. *P<0.05; **P<0.01; ***P < 0.001.

**Figure 5.**
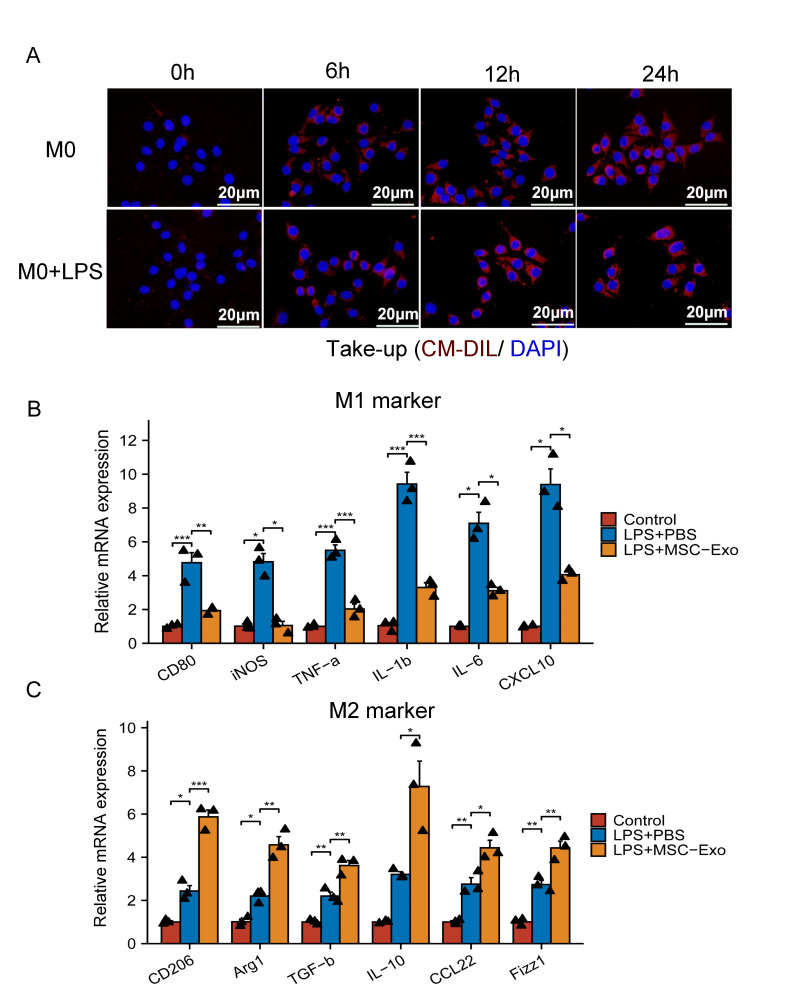
**MSC-Exos facilitate the polarization of macrophages to the M2 phenotype in an inflammatory environment**. **(A)** Representative images of the uptake of Dil-labeled exosomes (red) by M0 macrophages (DAPI, nucleus, blue; scale bar = 20 µm) and fluorescence uptake by LPS-stimulated M0 macrophages (DAPI, nucleus, blue; scale bar = 20 µm). (B, C) Gene expression profiles of M1 markers (CD80, iNOS, TNF-α, IL-1β, IL-6, and CXCL10) and M2 markers (CD206, Arg1, TGF-β, IL-10, CCL22, and Fizz1) in LPS-stimulated peritoneal macrophages after culturing with MSC-Exos or PBS for 48 h (n= 3; each black triangle represents one sample). The expression of genes of interest was normalized to that of GAPDH and reported as relative change. All data are presented as the mean ± SD. Statistical significance was determined using Welch one-way ANOVA with the Games-Howell *post hoc* test for CD80, IL-6, and CXCL10. The other groups were using one-way ANOVA followed by Tukey’s HSD *post hoc* test. *P<0.05; **P<0.01; ***P<0.001.

**Figure 6.**
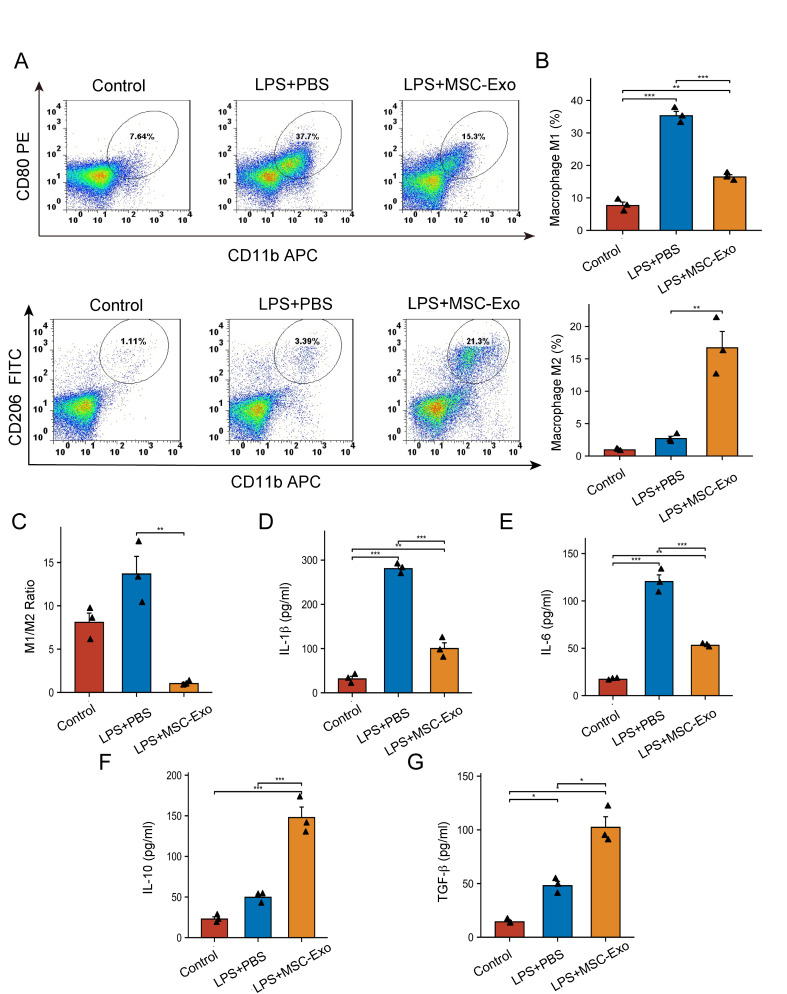
**MSC-Exos inhibited the inflammatory response by increasing the ratio of M2 macrophages to M1 macrophages *in vitro***. **(A)** Representative flow cytometry plots showing the percentages of M1 (CD11b+CD80+) and M2 (CD11b+CD206+) phenotypes in LPS-stimulated peritoneal macrophages after culture with MSC-Exos or PBS for 48 h (n=3). **(B)** Quantification of flow cytometry data in (A) (n=3; each black triangle represents one sample). **(C)** The M1/M2 ratio was calculated by flow cytometry (n=3; each black triangle represents one sample). Statistical significance was determined using one-way ANOVA followed by Tukey’s multiple comparisons test for multiple groups. **(D-G)** Concentrations of the cytokines IL-1β (C), IL-6 (D), IL-10 (E), and TGF-β1 (F) in the supernatants of LPS-stimulated M0 macrophages after culture with MSC-Exos or PBS for 48 h (n=3; each black triangle represents one sample). All data are the mean ± SD. Statistical significance was determined using Welch one-way ANOVA with the Games-Howell *post hoc* test for IL-10 and TGF-β. The other groups were using one-way ANOVA followed by Tukey’s HSD *post hoc* test. *P<0.05; **P<0.01; ***P<0.001.

**Figure 7.**
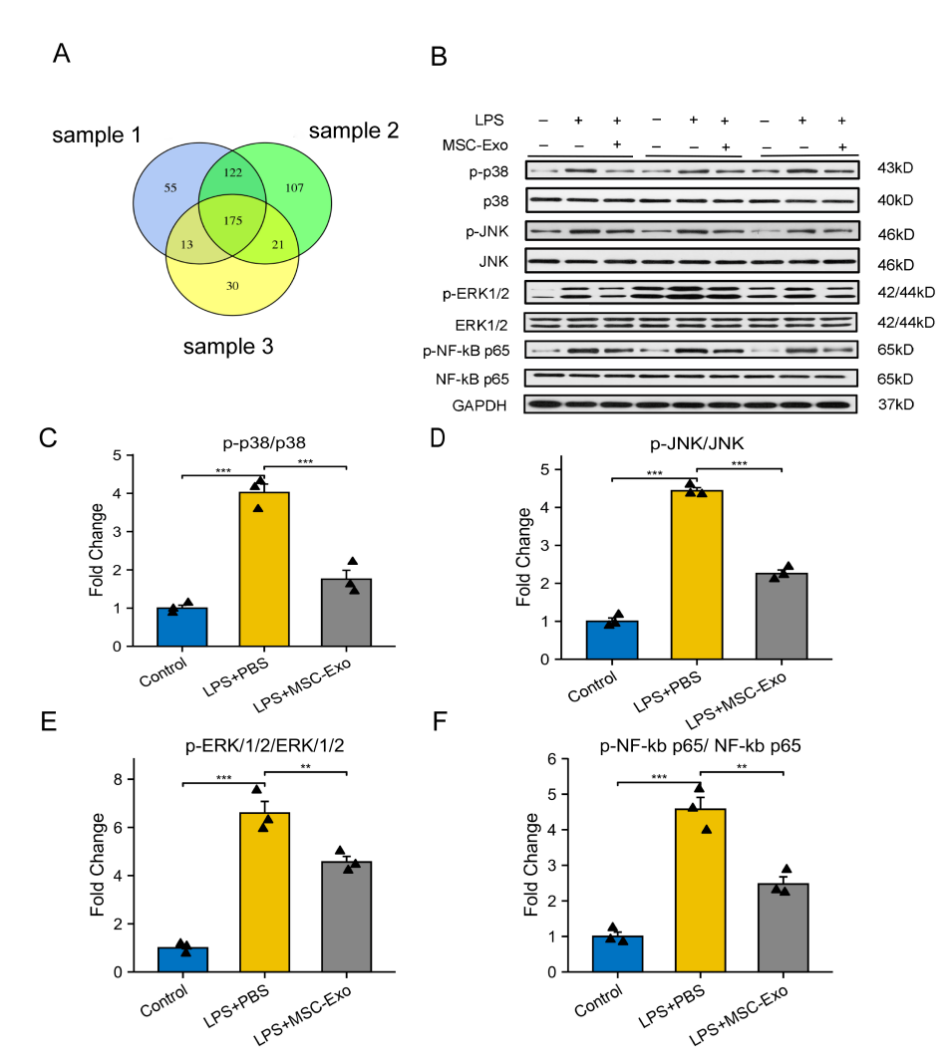
**MSC-Exos suppressed the activation of classical NF-κB and MAPK signaling in macrophages**. **(A)** Venn diagrams displaying the intersections of miRNAs in MSC-Exos (n=3). **(B)** Representative Western blotting images were collected to assess the levels of ERK1/2, p38, JNK, NF-κb and their phosphorylated forms (p-ERK1/2, p-p38, p-JNK, p-NF-κb) in macrophages under various circumstances (n=3). **(C-F)** Quantification of phospho-p38/p38 (C), phospho-JNK/JNK (D), phospho-ERK1/2/ERK1/2 (E), and phospho-NF κb p65/NF κb p65 (F) is shown (n=3; each black triangle represents one sample). All data are the mean ± SD. Statistical significance was determined using one-way ANOVA followed by Tukey’s HSD *post hoc* test. *P<0.05; **P<0.01; ***P<0.001.

**Figure 8.**
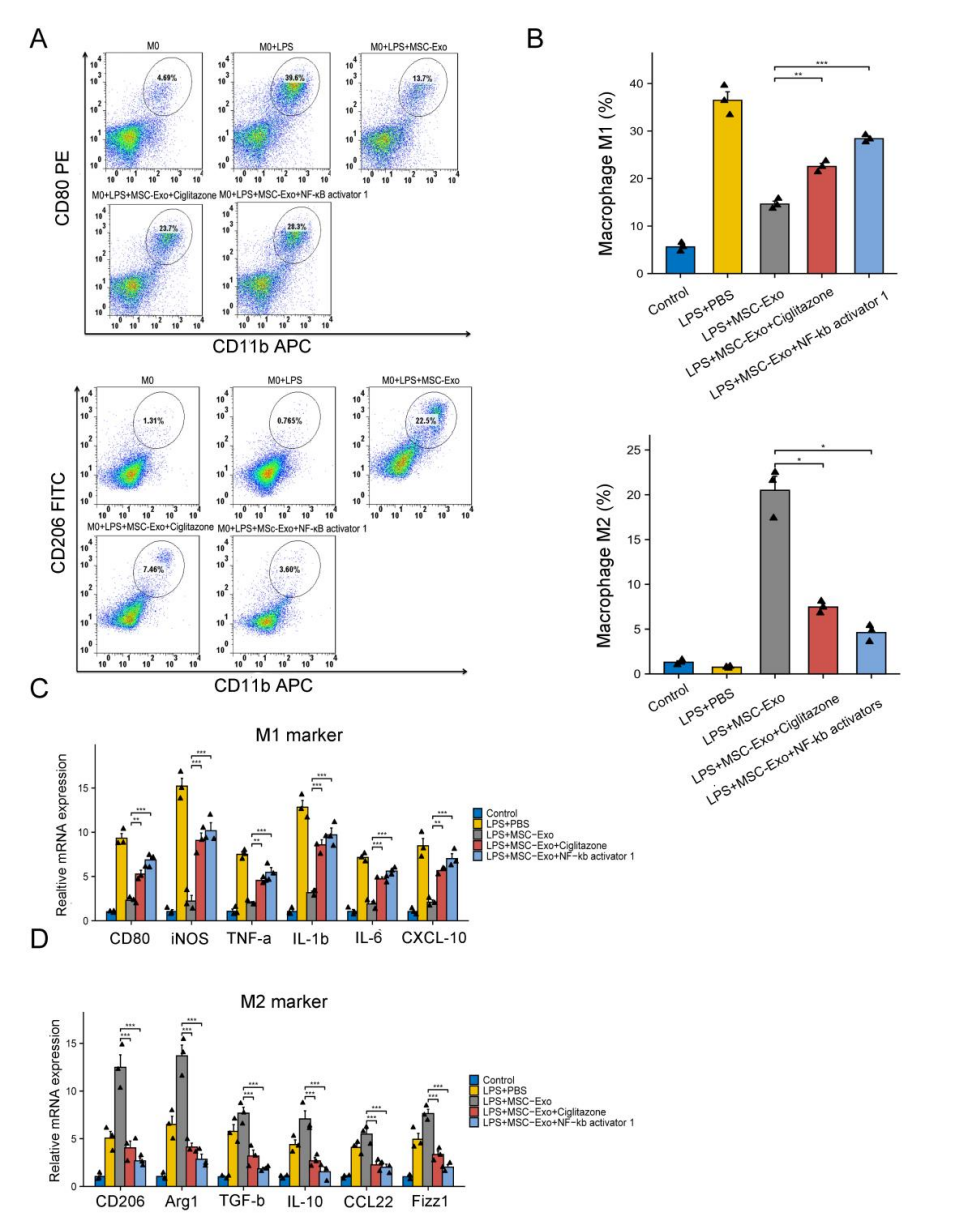
**MSC-Exos modulated macrophage polarization by mediating the p38 MAPK/NF-κB signaling pathway**. (A, B) Flow cytometry analysis and quantification of CD80+ macrophage percentage and CD206+ macrophage percentage under different treatments (n = 3; each black triangle represents one sample). Because of unequal variances, data with M2 were tested with Welch one-way ANOVA with the Games-Howell *post hoc* test. Statistical significance of M1 was determined using one-way ANOVA followed by Tukey’s HSD *post hoc* test. (C, D) qRT–PCR analysis of M1 marker (CD80, iNOS, TNF-α, IL-1β, IL-6, and CXCL10) and M2 marker (CD206, Arg1, TGF-β, IL-10, CCL22, and Fizz1) gene expression in macrophages under different treatments (n = 3; each black triangle represents one sample). All data are the mean ± SD. Statistical significance was determined using one-way ANOVA followed by Tukey’s HSD *post hoc* test. *P<0.05; **P<0.01; ***P<0.001.

Supplementary Table 1 shows the primer sequences used for *in vitro* experiments, and Supplementary Table 2 shows the primer sequences used for *in vivo* experiments.

### Exosomal miRNA microarray assay and bioinformatics analysis

We used the miRNeasy Mini kit (Qiagen, cat. No. 217004) to extract and purify total RNA from exosomes following the manufacturer’s instructions. Using the Agilent Bioanalyzer 2100 System’s RNA Nano 6000 Assay Kit, we assessed RNA concentration and purity (Agilent Technologies, CA, USA). According to the instructions of the manufacturer, we created sequencing libraries using the QIAseq miRNA Library Kit (Qiagen, Frederick, MD) and added index codes to assign sequences to each of the three samples. The library preparations were sequenced on an Illumina NovaSeq 6000 platform, with paired-end reads produced. After controlling the raw data and filtering repeats and ncRNAs (ribosomal RNA, transfer RNA, small nuclear RNA, and small nucleolar RNA), we aligned the sequencing reads to the miRbase and Human Genome (GRCh38) to identify recognized miRNAs. The transcript per million (TPM) value was obtained from the expression matrix of quantified miRNA UMI counts, and differential analysis was carried out using the EdgeR package. The consequences and sophisticated functioning of biological systems were investigated via the Kyoto Encyclopedia of Genes and Genomes (KEGG) database (www.genome.jp/kegg/). We tested the statistical enrichment of coexpressed genes in KEGG pathways utilizing an R script, and we calculated the p value using Fisher’s exact test.

### Establishment of the facial nerve injury rat model and experimental groups

The Peking Union Medical College Hospital Ethics Committee and the Ethics Committee of the Basic Medical Sciences, Chinese Academy of Medical Sciences have given their clearance for our work. The Experimental Animal Institute of the Chinese Academy of Medical Sciences (Beijing, China) provided adult male Sprague–Dawley rats of six to eight weeks, which were employed following the regulations of animal management of the Chinese Academy of Medical Sciences (kept in sterile conditions with a body weight of 280-320 g).

The following three groups of fifteen rats each were formed from the forty-five total animals: sham, PBS, and MSC-Exos. In summary, 40 mg/kg 1% pentobarbital sodium was intraperitoneally injected into the animals. Using mosquito forceps that were 1.5 mm in length, the left extracranial facial nerve main trunk was exposed and squeezed 0.5 cm from the stylomastoid foramen. Immediately after injury, the rats were given a local injection of either a 100 μL solution containing 100 µg of MSC-Exos or an equivalent volume of PBS. The sham group rats underwent surgery without damage or treatment.

Rats were sacraficed 1, 3, 7, and 14 days after injury. Before sacrifice, each rat underwent a facial nerve function behavioral analysis by two researchers who were blinded to the treatment status. Wang’s approach was used to test and rate facial nerve function. The three main evaluation factors were tip position (TP), vibrissae movement (VM), and blink reflex (BR) [22]. For BR measurement, a 5 mL syringe of gas was swiftly injected 2 cm from the damaged eye, and we observed the movement of the rat’s eyelids. For VM measurement, bilateral beard movement was tracked for 30 seconds. TP measurement was based on where the nose tip was located. In Supplementary Table 3, scoring guidelines are displayed. A successful model for facial nerve injury was established when a total score of three (3) or above was reached.

### Western blotting

Western blotting was performed using a standard protocol. Briefly, we extracted proteins from cells and treated them with radioimmunoprecipitation (RIPA) lysis and extraction buffer (Beyotime, P0013C). The protein concentration was determined using the BCA technique. We employed SDS–PAGE to isolate equal amounts of protein, which was then transferred to PVDF membranes (Millipore) and treated with primary antibodies for one night at 4 °C before blocking with 5% bovine serum albumin. Next, the membranes were incubated with the secondary antibody for 1 h at room temperature. The primary antibodies used in the animal experiment were as follows: Arg-1 (Affinity, cat# DF6657), iNOS (Affinity, cat# AF0199), phospho-NF-κB P65 (Affinity, cat# AF 2006), phospho-P38 (Affinity, cat# AF4001), phospho-ERK1/2 (Affinity, cat# AF1014), and phospho-JNK (Affinity, cat# AF3319). The following primary antibodies were utilized in *in vitro* experiments: NF-κB Pathway Sampler Kit (Cell Signaling Technology, cat# 8242), P38 (Cell Signaling Technology, cat# 8690), ERK1/2 (Cell Signaling Technology, cat# 4695), Phospho-NF-κB Pathway Sampler Kit (Cell Signaling Technology, cat# 3033), Phospho-ERK1/2 (Cell Signaling Technology, cat# 4370), Phospho-JNK (Cell Signaling Technology, cat# 9255), Phospho-P38 (Cell Signaling Technology, cat# 4511), and JNK (Cell Signaling Technology, cat# 9252). The secondary antibodies used in WB were goat anti-rabbit IgG (H+L) HRP (MultiSciences, China; cat#70-GAR0072) and goat anti-mouse IgG (H+L) HRP (MultiSciences, China; cat#70-GAM0072). An ECL reagent (Millipore Corporation, Billerica, MA, USA) was used to detect antibody-antigen complexes, and we semi-quantified the density of protein bands using ImageJ. β-Actin (Cell Signaling Technology, cat# 4970) and GAPDH (Cell Signaling Technology, cat# 5174) were used as internal controls.

### Histological analysis

Hematoxilin and eosin (HE) was performed using standard protocols. After sacrifice using intraperitoneal injection of 0.6% sodium pentobarbital overdose, we collected injured sections and fixed them in 4% paraformaldehyde on days 1, 3, 7, and 14 after operation. The obtained tissues were slowly dehydrated, and paraffin embedded. We sectioned these tissues at 5 μm, stained them with HE, and observed them under light microscopy.

### Immunofluorescence analysis

To do the immunofluorescence (IF), paraffin section was de-paraffinized using Xylene and rehydrated with gradient (100%, 95%,70%,0%) ethanol solution. IF was performed with standard procedures. Briefly, we processed tissue slides before blocking with normal goat serum. We then treated the slides with a fluorochrome-labeled secondary antibody for one hour after exposing them to primary antibodies overnight at 4 °C. The primary antibodies were MBP (cat# bs-0380R, Bioss, China), S100 (cat# 5292, Cell signaling Technology), and NF200 (cat# 18934-1-AP, Proteintech). The following antibodies were used for IF: Alexafluor 488 goat anti-rabbit secondary antibody (cat# MD912526, MDL, China) and DAPI (cat# C1005, Beyotime). DAPI was used to stain the nuclei for 10 min. Afterward, fluorescence observation was performed using a fluorescence microscope. ImageJ (NIH Image) was used for quantitative analysis.

### Transmission electron microscopy

We fixed the samples in 2.5% glutaraldehyde (pH 7.4) for 2 h. After rinsing three times with 0.1 M phosphate buffer (pH 7.2) and fixing in 1% osmic acid at 4 °C for 2 h, the samples were then dehydrated in an ethanol gradient in a graded sequence. The samples were then placed in a polymerization model and implanted in Epon-Araldite resin to allow for penetration. We created and collected the ultrathin segment for microstructure research after the section was used for placement. Counterstaining with 3% uranyl acetate and 2.7% lead citrate was then performed. The axon diameter was divided by the diameter of the enclosing myelin sheath, and g-ratios were calculated after axons were studied using an HT7800 transmission electron microscope. G-ratio statistics involved pooling all of the observations from each animal and comparing the mean number of myelinated and unmyelinated axons.

### Enzyme-linked immunosorbent assay

*In vivo*, blood was collected by eyeball extubation and then subjected to centrifugation for serum separation. We utilized serum for ELISA to estimate the levels of proinflammatory cytokines, including IL-1β (cat# MD132598, MDL, China) and IL-6 (cat# MD 132597, MDL, China), and anti-inflammatory cytokines, including TGF-β (cat# MD132599, MDL, China) and IL-10 (cat# MD132596, MDL, China), following the manufacturer’s instructions. Serum samples were isolated 7 days after facial nerve injury. A microplate reader (Bio-RAD, Model 680) was utilized to measure the absorbance at 450 nm and determine optical density (OD) values.

*In vitro*, the cell culture supernatant was subjected to ELISA following the manufacturer’s instructions (Human IL-1β ELISA kit, EHC002b, QuantiCyto, China; Human IL-6 ELISA kit, EHC007, QuantiCyto, China; Human TGF-β1 ELISA kit, EHC107b; Human IL-10 ELISA kit, EHC009, QuantiCyto, China). We used a microplate reader to measure the OD values for ELISAs.

### Statistical analyses

Data are shown as mean±SD or median and interquartile range (IQR).

For multi-group comparisons, the data met the assumptions of homogeneity of variance and normality, and statistical significance was determined using one-way ANOVA followed by Tukey’s HSD *post hoc* test. Statistical analysis of data not satisfying homogeneity of variance was performed using Welch’s ANOVA followed by Games-Howell *post hoc* test. Besides, data were analyzed by two-way repeated measures analysis of variance (ANOVA, factors: treatment and time) followed by univariate tests of simple main effects with Bonferroni correction for post hoc comparisons. Data did not follow a normal distribution; a non-parametric Mann-Whitney test was performed. All statistical analyses were conducted in R (version 4.2.1), and we utilized the ggplot2 package (version 3.3.6) for data visualization. Statistically significant differences were defined as *p< 0.05; **p<0.01; ***p<0.001. Randomization and group allocation were performed according to the animal weights. All the animals were included in the analysis.

## RESULTS

### Characterization of hAdMSCs

To characterize the hAdMSC population, we examined the phenotypic characteristics of hAdMSCs by flow cytometry analysis. Their ability to differentiate into various lineages was assessed by adipogenic and osteogenic differentiation (Supplementary Fig. 1). Their pluripotency was evaluated by performing multiple lineage differentiation, including by osteogenesis and adipogenesis. Oil red O staining was employed to evaluate adipogenic differentiation. Oil red O staining showed prominent intracellular lipids on day 12 (Supplementary Fig. 1A). Osteogenic differentiation was detected using ALP and ARS staining. ALP staining on day 6 and ARS staining and quantification on day 12 were carried out (Supplementary Fig. 1B and C). Moreover, according to flow cytometry, the surface-positive markers included CD29, CD73, CD44, CD90, and CD105, while the surface-negative markers included CD106, CD206, CD34, CD45, and HLA-DR (Supplementary Fig. 1D). The MSC properties and pluripotency of hAdMSCs were confirmed by the abovementioned results.

### Characterization of MSC-Exos

MSC-Exos were purified using ultracentrifugation from the hAdMSC supernatant and identified using WB, TEM, and NTA. The exosome sample appeared to be enriched in the exosome biomarkers Alix, CD81, HSP70, and Tsg101 according to the WB results in Figure 1A; however, calnexin, which is expressed on the endoplasmic reticulum membrane, was much less abundant in the exosome sample than in the cell lysate sample. The majority of isolated exosomes displayed characteristic lipid bilayer membrane encapsulation, cup- or sphere-shaped morphology, and exosome isolation based on TEM imaging (Fig. 1B). The outcome of NTA showed that the plasma exosome width varied between 50 and 200 nm (Fig. 1C). The aforementioned characteristics therefore suggested that the particles from MSCs that we obtained in our studies were exosomes.

### MSC-Exo administration promoted functional behavioral recovery after FN injury

In this study, we investigated whether MSC-Exos could have beneficial effects on FN injury by first evaluating the functional recovery of PBS- and MSC-Exo-treated rats utilizing facial expression score. All the rats in the sham group had no facial paralysis and the facial expression score was 0. A higher score indicates more severe facial paralysis symptoms. Facial paralysis is diagnosed when a total score is greater than 3 points. In Figure 1D, we compared the facial expression scores of the PBS group and MSC-Exos group. SD rats in the MSC-Exo group (median=2, IQR=0.5) had superior facial functional improvement compared with rats in the PBS group (median=4, IQR=0.5) at 14 days after injury (p<0.05).

Results of HE staining under the optical microscope are shown in Fig. 2A. In the sham operation group, the nerve tissue had orderly arranged fibers and a unified myelin sheath structure, which was consistent at four postoperative time points. In the PBS and MSC-Exo groups, the facial nerve fiber distribution was disordered, axons were swollen or missing, inflammatory cells infiltrated, and interstitial edema with hemorrhage was severe at 1 and 3 days. At 7 days, compared to the PBS group, the nerve in the MSC-Exo group presented a more regular distribution and less axonal swelling and vacuolation, the interstitial edema with hemorrhage was significantly alleviated, and more Schwann cells and fewer inflammatory cells were arranged between the fibers in the MSC-Exo group. At 14 days, the pathomorphological changes in the MSC-Exo group were similar to those in the sham operation group. In addition, at 14 days after FN injury, TEM suggested that myelinated axons in the MSC-Exo group had thicker myelin sheaths than that of the control group, which were similar to that of the sham operation group, as indicated by the remarkable decrease in the G-ratio (myelin inner diameter/outer diameter) (P<0.001) (Fig. 2B).

For the regrowth of axons and the evaluation of nerve myelination, neurofilament 200 (NF200, represents axonal growth), S100 (the specific marker of Schwann cells), and myelin basic protein (MBP, a marker of myelination) were involved in the IF assay. The images in Figure 3 were taken at 1, 3, 7, and 14 days, revealing a progressive process of repair and regeneration. The staining results illustrated that the MBP-, NF200-, and S100-positive areas were dense and neatly distributed in the sham group (Fig. 3A). We observed that the average optical density value (IOD/area) of NF200 on days 7 and 14 was remarkably higher in the MSC-Exo group than in the PBS group (p<0.001, Fig. 3B). The expression of MBP and S100 showed the same trend as the expression of NF200 (Fig. 3A). The statistical results of positive cell percentages of NF200, MBP and S100 are shown in Figure 3B. These data indicated that MSC-Exos enhanced axonal regeneration and myelination in FN injury for repair.

### MSC-Exos decreased the M1 macrophage number and enhanced macrophage transformation toward the M2-like phenotype under FN injury

The ability of macrophages to flip between M1 and M2 phenotypes in response to damage is well known. Thus, we wondered whether MSC-Exos could polarize macrophages toward the M1 or M2 phenotype under FN injury. On day 7 after injury, the tissue was removed, and the cells were examined using Western blotting and qRT–PCR (Fig. 4).

Western blotting analysis showed that iNOS was significantly reduced in the MSC-Exo group compared with the PBS group (p<0.01), whereas Arg1 expression was significantly upregulated (p<0.001, Fig. 4A and B). M1 and M2 qRT–PCR analysis also showed that M1 markers (CD80, iNOS, IL-1β, TNF-α, IL-6, and CXCL10) were obviously decreased in the MSC-Exo group, while M2 markers, such as CD206, Arg-1, TGF-β, IL-10, CCL22, and Fizz1, were also decreased (Fig. 4C and D), which agreed with the Western blotting analysis.

Furthermore, blood samples were taken day 7 postinjury, and serum was collected for inflammatory cytokine detection by ELISA. The results showed that the levels of proinflammatory secreted cytokines (IL-1β and IL-6) were obviously reduced in MSC-Exo-treated SD rats compared to PBS-treated rats (p<0.001). Additionally, the anti-inflammatory secreted cytokines (IL-10, TGF-β) were increased following MSC-Exo treatment (p<0.001, Fig. 4E-H). Our findings showed that MSC-Exos reduced the localized and systemic inflammatory response by polarizing macrophages away from the M1 phenotype and toward an M2-like state in response to FN damage.

### MSC-Exos suppressed the inflammatory response by increasing the ratio of M2-M1 polarization in vitro

To further determine the effects of MSC-Exos on the function of macrophages, we examined whether exosomes could enter macrophages under inflammatory (LPS induced) and noninflammatory (non-LPS induced) conditions. The results showed that regardless of whether it was in an inflammatory environment (LPS induced) or not, CM-DIL (red)-labeled MSC-Exos were readily internalized into macrophages, localized in the cytoplasm within 6 h and their levels peaked after 24 h (Fig. 5A).

We conducted qRT–PCR to evaluate the relative gene expression of pro- and anti-inflammatory genes. The expression level is relative to the GAPDH gene. The results showed that in comparison to the PBS and LPS groups, the relative levels of CD80, iNOS, IL-1, TNF-, IL-6, and CXCL10 in the MSC-Exo groups dramatically decreased (Fig. 5B). In comparison to the PBS and LPS groups, the relative levels of CD206, Arg-1, TGF-, IL-10, CCL22, and Fizz1 in the MSC-Exo groups were remarkably higher (Fig. 5C). In addition, flow cytometry analysis revealed that the MSC-Exo group’s ratio of M2 macrophages was considerably higher than that of M1 macrophages (p<0.01, Fig. 6A and B). Moreover, the MSC-Exo group had a lower M1/M2 macrophage ratio (p<0.01, Fig. 6C) compared to the other groups.

After 48 h, the concentrations of proinflammatory secreted cytokines (IL-1β, IL-6) and anti-inflammatory secreted cytokines (IL-10, TGF-β) in the culture supernatant were determined by ELISA. In comparison to the PBS and LPS groups, IL-1β and IL-6 secretion was significantly decreased in the MSC-Exo group (p<0.001, Fig. 6D and E). In contrast, we found that the MSC-Exo group exhibited significantly increased IL-10 and TGF-β secretion compared with the PBS and LPS groups (p<0.01, Fig. 6F and G). All of the aforementioned data demonstrated that MSC-Exos could decrease the ratio of M1/M2 macrophages, which indicates their therapeutic potential in diseases related to inflammation.

### MSC-Exos partially regulated macrophage polarization by mediating the MAPK/NF-kb signaling pathway

miRNA sequencing was applied to analyze the mechanism by which MSC-Exos regulate the shift in macrophage phenotype. The Venn diagram shows the common and unique miRNAs between the three MSC-Exo samples, revealing 175 intersecting miRNAs (Fig. 7A). The 100 top-expressed miRNAs are shown in Supplementary Table 3. Then, we subjected the groups of miRNAs to KEGG pathway analysis to understand their biological functions. The KEGG results showed that these miRNAs were involved in multiple significant pathways, including the MAPK pathway (Supplementary Fig. 2A). In addition, the literature shows that MAPK activation can occur upstream of NF-κB signaling.

To determine whether MSC-Exos were impacting the M1-M2 macrophage shift by inhibiting the MAPK and NF-κB pathways, the expression of MAPK and NF-κb pathway-associated proteins was measured, such as the total protein levels of ERK1/2, p38, JNK, NF-κb and their phosphorylated forms (p-ERK1/2, p-p38, p-JNK, p-NF-κb), which were measured in macrophages under various circumstances by WB analysis (Fig. 7B). The results suggested that the phosphorylation levels of p38, ERK1/2, JNK, and NF-κb were upregulated by LPS stimulation and decreased by MSC-Exo treatment, whereas the total protein levels remained unchanged; this result indicated that MSC-Exos could suppress the phosphorylation of the MAPK/NF-κB pathway (p<0.01, Fig. 7C-F). To further confirm the in vitro findings, we also verified that the MAPK/NF-κB pathway was phosphorylated *in vivo* (Supplementary Fig. 2B). The WB results implied that the phosphorylation levels of p38, ERK1/2, JNK, and NF-κb were decreased by MSC-Exo treatment (p<0.05, Supplementary Fig. 2C-F). Importantly, in the MAPK pathway, the results showed that MSC-Exo treatment reduced the phosphorylation level of p38 the most (p<0.001).

A p38 MAPK agonist (ciglitazone) and an NF-κb agonist (NF-κb activator 1) were added together with MSC-Exos and incubated with macrophages stimulated by LPS for 48 h to further confirm that the regulatory action of MSC-Exos on macrophages occurred via the p38 MAPK/NF-κb pathway.

Flow cytometry was utilized to analyze the M1 and M2 markers for each group (Fig. 8A). The M1 macrophage percentage increased in the MSC-Exos with Ciglitazone and NF-κb activator 1 group (p<0.01) and decreased in the MSC-Exos with Ciglitazone and NF-κb activator 1 group compared to the MSC-Exo group (p<0.001); this result indicated that activating the p38 MAPK/NF-κb signaling pathway partially blocked the repolarization function of MSC-Exos (Fig. 8B).

Additionally, qRT–PCR analysis illustrated that proinflammatory gene expression (CD80, iNOS, IL-1β, TNF-α, IL-6, and CXCL10) was to some degree increased when the p38 MAPK/NF-κb signaling pathway was activated in the MSC-Exos with Ciglitazone and NF-κb activator 1 group (p<0.01, Fig. 8C); meanwhile, anti-inflammatory gene expression (CD206, Arg-1, TGF-β, IL-10, CCL22, and Fizz1) was partly decreased (p<0.001, Fig. 8D). In summary, the obtained results implied that the repolarization of macrophages was, at least partially, regulated by inhibiting the p38 MAPK/NF-κb signaling pathway via MSC-Exos.

## DISCUSSION

In social interactions, facial expressions are crucial. Peripheral facial nerve injury and facial paralysis can be caused by inflammation, trauma, tumor removal, and surgical intervention [2, 23]. This common but severe disorder leaves patients with functional, esthetic, mental, and psychological problems [24]. Even while surgical methods and synthetic nerve conduits have made substantial strides, full regeneration after FN injury is still far from ideal [25, 26]. In this study, we first showed that MSC-Exos had healing properties based on their promotion of axonal regeneration and myelination following FN injury and the decrease in the localized and systemic inflammatory response. Our research has also shown that, in *in vivo* and in vitro experiments, MSC-Exos efficiently changed macrophage polarization toward the M2 rather than the M1 phenotype, which may have the effect of reducing inflammatory cascades and promoting subsequent reparative activities. Additionally, we showed that the p38 MAPK/NF-κB pathway was used by MSC-Exos to modulate the macrophage phenotype.

It takes a coordinated effort by nonneuronal cells, such as immune cells, and a strong regenerative response from wounded axons for a nerve to heal and regenerate after injury [27]. The distal section of wounded PNS axons gradually degenerates (Wallerian degeneration), which is caused by the destruction of both myelin and axons [28]. Schwann cell dedifferentiation and immune response activation are associated with Wallerian degeneration, which is crucial for starting the repair response [29]. Meanwhile, excessive immune responses may promote local scar formation and hyperplasia, which have a direct impact on conduction recovery following nerve injury. They may also induce the expression of inflammatory mediators to surround the injured tissue. Therefore, creating a microenvironment that supports an appropriate inflammation and regeneration level could be a critical objective for the treatment of FN injury. The strong immunosuppressive and anti-inflammatory properties of MSCs have been considered a possible treatment for FN injury during the past 20 years [9, 30]. Bone marrow mesenchymal stem cells (BMMSCs), human umbilical cord mesenchymal stem cells (hUCMSCs), and adipose-derived mesenchymal stem cells (AdMSCs) are the MSCs that are most frequently used in clinical practice [31]. Because AdMSCs are readily available and have a large population of mesenchymal stem cells, we decided to use them as the source of stem cells in this work [32]. There is still some uncertainty regarding how MSCs help with nerve healing. However, several researchers believe that rather than through transdifferentiation, the therapeutic impact of transplanted MSCs is more likely to be caused by a cellular paracrine process [33-35].

Exosomes are biological nanoparticles with lipid bilayer encasements ranging from 30 to 200 nm in size and are present in practically all bodily fluids, including blood, breast milk, ascites, saliva, and urine [14]. They have started to be recognized as significant paracrine system mediators. Due to their various benefits over MSCs, these nanoparticles have favorable therapeutic effects that are comparable to those of MSCs and may be effective tools for cell-free therapy [36]. Exosomes have been linked to pathological and physiological circumstances, including malignancies, tissue fibrosis, and neurological disorders, according to recent investigations [37-39]. Exosomes carry horizontally transferable cellular signaling molecules (proteins, lipids, lncRNAs, circRNAs, mRNAs, miRNAs, and DNA) that facilitate communication between cells [40]. Exosomes produced from MSCs have been demonstrated to have positive influences in numerous disease models, such as those for cancer [41, 42], hepatic fibrosis [43, 44], spinal cord injury [11, 45], and myocardial infarction [13, 46]. Importantly, the utilization of MSC-derived exosomes in therapeutic methods for regenerating peripheral nerves is possible [47]. However, rather than using the facial nerve, the majority of these studies employ the sciatic nerve. An accurate representation of the facial nerve's complicated topographic architecture and the clinical difficulties associated with synkinesis cannot be achieved using a sciatic nerve model [9]. Few studies have been performed to date on the potential impact of MSC-Exos on FN regeneration following injury and based on inflammation reduction. In our study, we unequivocally established that MSC-Exos support successful axonal regrowth and myelination following FN injury, and we also showed that the therapeutic effects of MSC-Exos were strongly correlated with their ability to control inflammation; furthermore, the effects persisted throughout the acute inflammation and healing phases.

Macrophages are highly malleable, heterogenic immune cells that are crucial for peripheral nerve injury recovery and act by removing waste and controlling the microenvironment [4, 48]. A prior study demonstrated that Wallerian degeneration and subsequent regeneration in peripheral nerve damage are negatively impacted by total macrophage ablation via pharmacologic or genetic methods [49]. According to a different study, inflammation caused by macrophages is the mediator of Schwann cell dedifferentiation [50]. M1 and M2 are two macrophage types. Compared to M2 macrophages, which are immunosuppressive cells that facilitate tissue remodeling and repair and participate more in the regenerative process, M1 macrophages are cells that are proinflammatory, trigger immunological responses and are more implicated in Wallerian degeneration [4, 7]. Many studies have demonstrated the potential for research on macrophage M2 polarization to provide unique and useful treatment strategies to enhance peripheral nerve damage regeneration [51-53].

Recent research has shown that MSC-Exos can control macrophage plasticity to help them polarize into the anti-inflammatory phenotype, which inhibits the production of proinflammatory cytokines [13, 54]. In prior work, a macrophage depletion model was used to demonstrate that the primary downstream target of MSC exosomes during cardiac ischemia/reperfusion was macrophages [13]. Additionally, another study revealed that MSC-Exos promote the polarization of microglia—the central nervous system's analog of macrophages—to an anti-inflammatory M2 phenotype [55]. Our research has further proven that MSC-Exos successfully induces M2 polarization and suppresses M1 polarization in FN-injured rats, as well as in vitro. By changing the phenotype of macrophages, MSC-Exos reduced proinflammatory cascades and boosted subsequent reparative actions. Moreover, according to the ELISA results in the rat model after FN injury, we hypothesize that these effects are not confined to local responses but rather can influence the systemic inflammatory response. In our work, we also found that MSC-Exos could enhance Schwann cell proliferation and differentiation, thereby promoting myelination after FN injury. Macrophages may participate in these pathways, although the mechanisms remain undefined.

To investigate the underlying mechanism of the suppressive effect of MSC-Exos on macrophage polarization, we performed microRNA (miRNA) sequencing analysis and bioinformatics analysis. We found enrichment of miRNA target genes in the MAPK signaling pathway, which is consistent with results of a previous study. Furthermore, it is recognized that NF-κB signaling is essential for triggering inflammatory reactions, and new studies suggest that activating MAPK can trigger inflammation by upstream activation of canonical NF-κB signaling [56]. Hence, we also chose to validate NF-κB activation. According to a recent study, activating the MAPK/NF-κB signaling pathway is necessary for M1 macrophage polarization, and inhibiting this signaling pathway could prevent M1 macrophage polarization and encourage M1-M2 polarization. In this study, the results of a WB assay indicated that hAdMSC-Exos suppressed the phosphorylation of the ERK1/2, p38, JNK MAPK and NF-κB pathways in lipopolysaccharide-stimulated THP-1 cells (M1 phenotype), inhibited the secretion of inflammatory factors and enhanced the polarization of macrophages to the M2 phenotype in vitro. Rescue experiments were also utilized to detect the changes in macrophage polarization and further verify that MSC-Exos exerted their effect through regulation of the p38 MAPK/NF-κB pathway, which was significantly suppressed in the WB assay. Importantly, we also demonstrated the mechanism *in vivo* in animal models. These findings offer important information about the effects of MSC-derived exosomes on macrophage activity, even though they may not entirely reflect changes in the functional status of macrophages *in vivo*. Furthermore, regarding the KEGG pathways, the “cGMP-PKG signaling pathway” was also enriched. The resulting NO increase in neurons activates cGMP and protein kinase G (PKG) signaling and affects multiple cellular processes, including neurotransmission, cell metabolism, long-term potentiation and depression, and neuropathic pain [57]. In addition, the “Wnt signaling pathway”, the “Notch signaling pathway”, the “Hippo signaling pathway”, and the “Rap1 signaling pathway” were also included in the KEGG analysis results. Although these pathways were not significantly enriched (p>0.05), they were also involved in inflammation and nerve regeneration. In addition, these pathways can also impact the MAPK signaling pathway. For instance, the Notch signaling pathway is a source pathway, which impacts the MAPK signaling pathway through the Notch interface gene [58]. Altogether, these results provide a reference for future studies.

This study has some limitations. First, we did not systemically deplete macrophages. Using clodronate liposomes to deplete macrophages impaired the benefits of MSC-Exo, indicating that macrophages were required for the nerve repair effects of MSC-Exo therapy. Second, the limitation of this study lies in the absence of a detailed mechanism for the treatment of FN injury by exosomes. Further research should focus on different metabolites or RNAs in exosomes, which can act as specific targets, to reveal the more detailed underlying mechanism, provide feasible treatment options, and identify more accurate biomarkers. Of course, exosome-mediated treatment has its own limitations, such as complex and cumbersome exosome extraction procedures and a limited quantity of exosomes[59]. Furthermore, another key challenge in exosome research and development is exosome heterogeneity because each exosome is unique in its molecular and biochemical composition[60, 61]. Overall, the function and use of stem cell-derived exosomes is still in its infancy, and the precise function of exosomes remains largely unknown. Therefore, there is a critical need to explore the functional roles of exosomes and their precise components that are critical for therapeutic delivery in follow-up studies.

In conclusion, our research shows that MSC-Exos promote FN regeneration and suppress inflammation after injury by increasing the ratio of M2/M1 polarization by blocking the phosphorylation of the p38 MAPK/NF-κB signaling pathway. The hope is that cell-free therapies could eventually result in the repair and regeneration of the damaged FN, and MSC exosome therapy for FN injury could be a safer, less expensive, and more successful treatment option. However, much work still has to be done in scientific and clinical studies.

## Supplementary Materials

The Supplementary data can be found online at: www.aginganddisease.org/EN/10.14336/AD.2023.0719-1.


