# Supplementary material for: Mesenchymal Stem Cell-Derived Exosomes Promote Recovery of The Facial Nerve Injury through Regulating Macrophage M1 and M2 Polarization by Targeting the P38 MAPK/NF-Κb Pathway
Source: Aging Dis. 2024 Apr 1;15(2):851–68. doi: 10.14336/AD.2023.0719-1 (PMC10917525; doi:10.14336/AD.2023.0719-1)
Supplement: Supplementary file 1 [file AD-15-2-851-s.pdf]

SUPPLEMENTARY DATA

Mesenchymal Stem Cell-Derived Exosomes Promote Recovery of The Facial Nerve Injury through Regulating Macrophage M1 and M2 Polarization by Targeting the P38 MAPK/NF-Kb Pathway

Ruoyan Xue, Mengyao Xie, Zhiyuan Wu, Shu Wang, Yongli Zhang, Zhijin Han, Chen Li, Qi Tang, Liping Wang, Di Li, Shihua Wang, Hua Yang, Robert Chunhua Zhao

© 2023. Xue R et al. Published online at http://www.aginganddisease.org/EN/10.14336/AD.2023.0719-1

SUPPLEMENTARY DATA

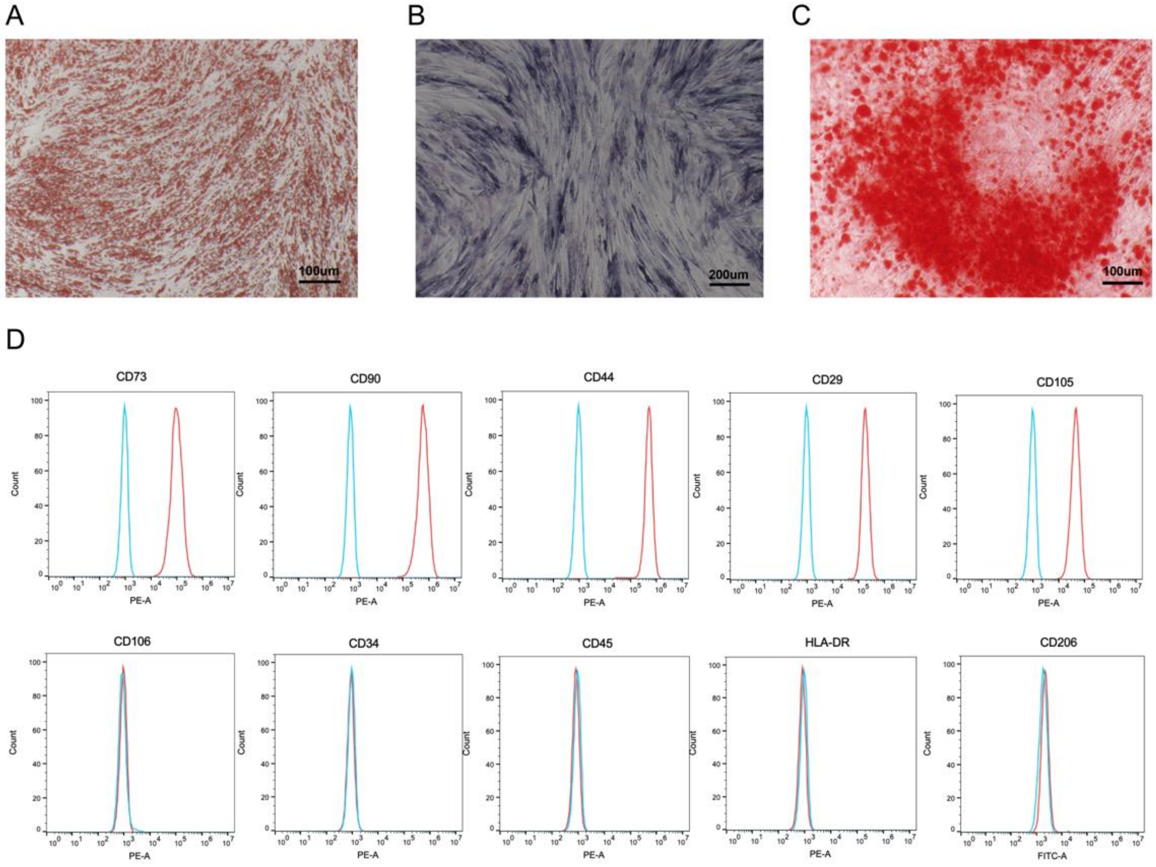

Supplementary Figure 1. Characterization of hAdMSCs. A: MSC adipogenic differentiation was demonstrated by Oil red O staining (scale bar=100 µm). B: MSC osteogenic differentiation was demonstrated by alkaline phosphatase (scale bar=200 µm). C: MSC osteogenic differentiation was demonstrated by Alizarin Red staining (scale bar=100 µm). D: Flow cytometry analysis found that the MSC markers CD29, CD44, CD73, CD90, and CD105 were positive, and CD34, CD45, CD106, CD206, and HLA-DR were negative.

© 2023. Xue R et al. Published online at http://www.aginganddisease.org/EN/10.14336/AD.2023.0719-1

SUPPLEMENTARY DATA

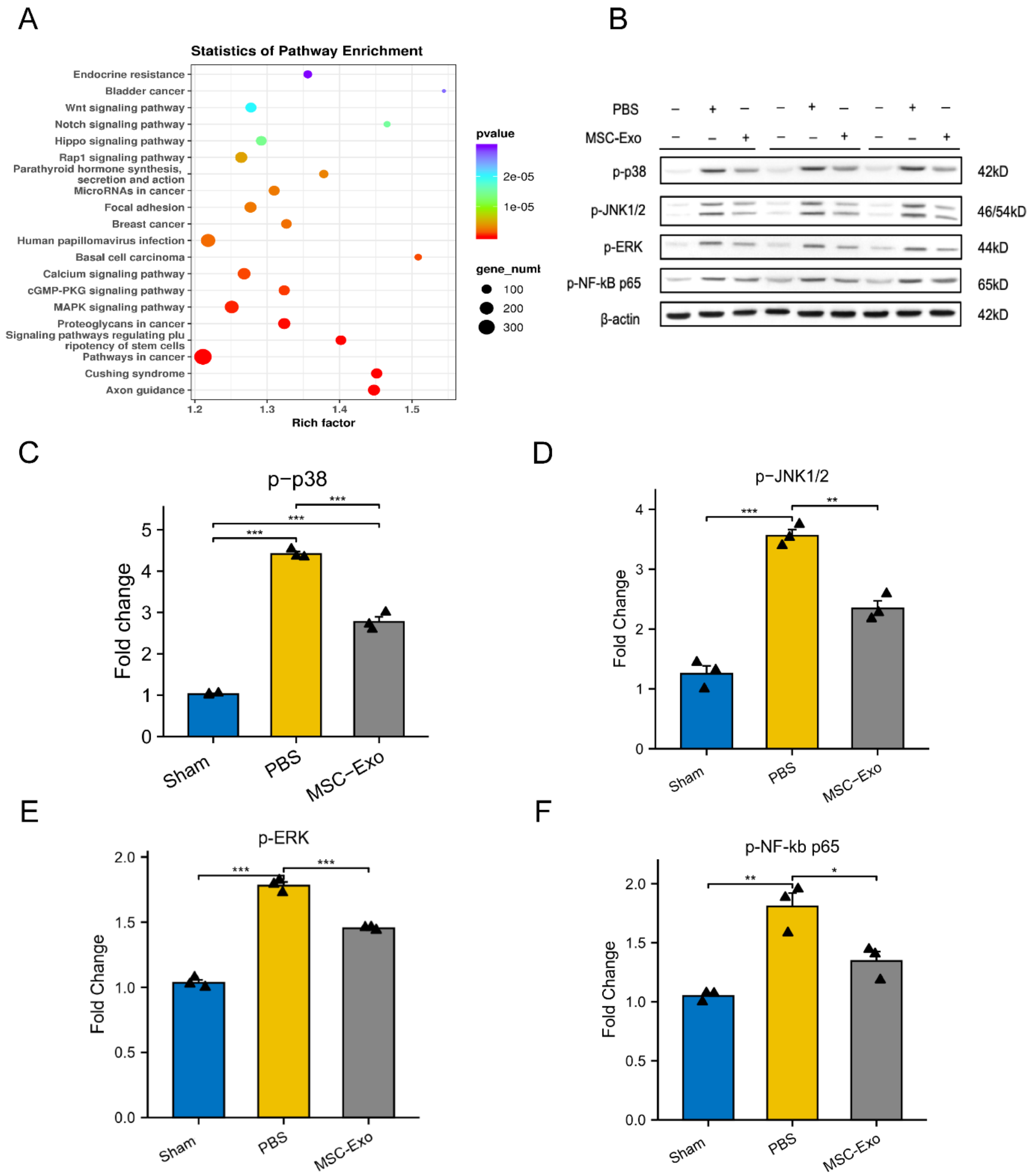

Supplementary Figure 2. MSC-Exos suppressed the activation of classical NF-κB and MAPK signaling in vivo. A: KEGG pathway enrichment analysis. The abscissa is the gene ratio, and the ordinate is the pathway name. The node size represents the number of genes in the enriched pathway. The node color represents -log10 (*p* value). B: Representative Western blot images to assess the levels of phosphorylated forms (p-ERK1/2, p-p38, p-JNK, p-NF-κb) in the facial nerves of rats treated with PBS or MSC-Exos 7 days after injury (n=3). C-F: Quantification of phospho-p38 (C), phospho-JNK1/2 (D), phospho-ERK (E), and phospho-NF-κb p65 (F) is shown (n=3). All data are the mean ± SD. Statistical significance was determined using one-way ANOVA followed by Tukey's HSD *post hoc* test. *P<0.05; **P<0.01; ***P<0.001.

© 2023. Xue R et al. Published online at http://www.aginganddisease.org/EN/10.14336/AD.2023.0719-1

SUPPLEMENTARY DATA

Supplementary Table 1. Sequences of qRT-PCR primers in vitro

Target gene	Primer	
TNF-α	F	5’-CTCATCTACTCCCAGGTCCTCTTC-3’
	R	5’-CGATGCGGCTGATGGTGTG-3’
IL-1β	F	5’-CGAATCTCCGACCACCACTA-3’
	R	5’-AAGCCTCGTTATCCCATGTGT-3’
IL-6	F	5'-AGGGCTCTTCGGGAAATGT-3'
	R	5'-GAAGAAGGAATGCCCATTAACAAC-3'
CXCL10	F	5'-CGCTGTACCTGCATCAGCAT-3'
	R	5'-TGCATCGATTTTGCTCCCCT-3'
CD206	F	5'-TTCGGACACCCATCGGAATTT-3'
	R	5'-CACAAGCGCTGCGTGGAT-3'
IL-10	F	5'- AACAAGAGCAAGGCCGTGG-3'
	R	5'-GAAGATGTCAAACTCACTCATGGC-3'
TGF-β	F	5'-CCCAGCATCTGCAAAGCTC-3'
	R	5'-GTCAATGTACAGCTGCCGCA-3'
CCL22	F	5'-AGGTATGGTGCCAATGT-3'
	R	5'-CGGCAGGATTTTGAGGTCCA-3'
CD80	F	5'-GGGAAAGTGTACGCCCTGTA-3'
	R	5'-GCTACTTCTGTGCCCACCAT-3'
iNOS	F	5'-AGCTGAACTTGAGCGAGGAG-3'
	R	5'-GGAAAAGACTGCACCGAAGA-3'
Arg1	F	5'-CAGATATGCAGGGAGTCACC-3'
	R	5'-CAGAAGAATGGAAGAGTCAG-3'
Fizz1	F	5'-CCGTCCTCTTGCCTCCTTC-3'
	R	5'-CTTTTGACACTAGCACACGAGA-3'
GAPDH	F	5'-GCACCGTCAAGGCTGAGAAC-3'
	R	5'-TGGTGAAGACGCCAGTGGA-3'

Supplementary Table 2. Sequences of qRT-PCR primers in vivo

Target gene	Primer	
β -actin	F	CCAGCCTTCCTTCTTGGGTA
	R	CAATGCCTGGGTACATGGTG
iNOS	F	TGCATGTGACTCCATCGACCC
	R	TGGACCCCATGCATAATTTGGAC
CD80	F	AGCAGTCCATACACCGAAT
	R	ATGTCGTATACAGTCCGGTTC
TNF- α	F	AGGAGGCAGATGCCAATGAG
	R	GGGCTGGTCATGGAAAGGA
IL-6	F	TGAGAAAAGAGTTGTGCAAT
	R	TTGTTTTCTGACAGTGCAT
IL-1 β	F	TGAAATAGCAGCTTTCGACAGT

© 2023. Xue R et al. Published online at http://www.aginganddisease.org/EN/10.14336/AD.2023.0719-1

SUPPLEMENTARY DATA

Target gene	Primer	
	R	AGATTTGAAGCTGGATGCTCT
CXCL10	F	TCATTCCTGCAAGTCTATCCTGT
	R	GACCTTCTTTGGCTCACCG
ARG-1	F	ATATCTGCCAAGGACATCGT
	R	CTCTTCCATCACTTTGCCAA
CD206	F	TATATGCCAAACACAGACCGAC
	R	TTTCTCTGCTTCGTGCCAT
TGF- β	F	ACTACGCCAAAGAAGTCACC
	R	ACTGCTTCCCGAATGTCTG
IL-10	F	GTGACAATAACTGCACCCAC
	R	CCTGCAGTAAGGAATCTGT
CCL22	F	CCAGGACTACATCCGTCACC
	R	CTGGGGTCAGCACAGATATCTCG
Fizzl	F	GGAAGACCCTCTCATGCAC
	R	TTAAGCACAGGCAGTTGCAAA

Supplementary Table 3. Facial expression score guideline

Criteria	Score	Evaluation
Blink reflex	0	No distinction between the two sides
	1	The injury side delayed movement
	2	The unclosed eyelid of the injury side
Vibrissae movement	0	No distinction between the two sides
	1	The injury side weakened exercise
Tip position	0	The middle tip of nose
	1	A contralateral nose

© 2023. Xue R et al. Published online at http://www.aginganddisease.org/EN/10.14336/AD.2023.0719-1
